# The influence of living conditions on adolescent girls’ health

**DOI:** 10.3402/qhw.v7i0.19059

**Published:** 2012-12-06

**Authors:** Margaretha Larsson, Annelie Johansson Sundler, Margaretha Ekebergh

**Affiliations:** 1 University of Skövde, Sweden; 2 Linnæus University, Växjö, Sweden

**Keywords:** Child, teenage, youth, female, women, personal circumstances, well-being, experiences, lifeworld dimensions, existential

## Abstract

Adolescence is described by the Swedish National Board of Health and Welfare as the healthiest period in life. However, adolescent girls differ in that they self-report that their health decreases with age. The aim of this hermeneutical study was to describe the meaning of living conditions in relation to adolescent girls’ health. Guided by principles of reflective lifeworld research, 15 interviews with adolescent girls were analysed. The result section consists of four narratives with their existential interpretations illustrating different ways of approaching living conditions and their meaning for health and well-being. The narratives are: Approaching everyday life in a balanced way—feeling harmonious; approaching everyday life with ambiguity—feeling confused; approaching everyday life as an intellectual project—striving for control; approaching everyday life as a struggle—feeling forlorn. In addition, a comprehensive understanding was developed by using the lifeworld dimensions: lived body, lived room, lived time, and lived relations. These dimensions may deepen the understanding of important parts of those living conditions which are meaningful for the girls’ health and well-being. By using the dimensions, complex living conditions have been explored and the meaning of different parts clarified. The girls’ thoughts and feelings are often ambiguous and sometimes contradictory, depending on the situation. The health of adolescent girls needs to be understood against the background of their experiences of living conditions. One way to support their health and well-being seems to be to supply them with forums where they can talk about their living conditions.

The Swedish Socialstyrelsen (2009) describes adolescence as the healthiest period in life. However, adolescent girls differ in that they self-report that their health decreases with age. During teenage years, the typical adolescent girl's ability to make choices and take responsibility for her own circumstances in life increases (Hwang & Nilsson, 2011). The environment in which an adolescent grows up may limit his or her potential to make appropriate personal choices and take a stand during the teenage years (Socialstyrelsen, 2009).

According to the World Health Organization (WHO) study, Health Behavior in School-aged Children, living conditions and lifestyle influence how adolescents self-report their health and well-being (Cavallo, Zambon, Borraccino, Raven-Sieberer, Torsheim & Lemma, 2006; Danielson, 2006; Statens Folkhälsoinstitut, 2011). These reports and other studies show that adolescent girls’ personal accounts of their health are influenced by elements in their environment, such as having friends (Johansen, Rasmussen, & Madsen, 2006), having support from the surrounding world (Callaghan, 2006), bullying, experiences in school, family structure, their ability to talk to their parents (Currie et al., 2008), disability, and health-compromising habits (Breidablik, Meland, & Lydersen, 2009). Some research has been conducted into the connection between adolescent girls’ health and the conditions under which they live. Connections between different factors can be seen, but little research has examined how an adolescent girl understands her own experiences of her living conditions. Johansson, Brunnberg, and Eriksson (2007) argue that relations with family and friends are most important for the mental health of adolescents. Furthermore, responsibility and performance are dynamic processes that also influence adolescent mental health (Landstedt, 2010). Tinnfält (2008) argues that all levels in society influenced the mental health among adolescents.

Health is a phenomenon with multiple meanings. In this study, health is understood in terms of the description of Dahlberg and Segesten (2010). They argue that health is an experience of feeling well and living a good life. Health is a feeling of being able to carry out smaller and bigger life projects. To be able to support the development of adolescent girls’ health processes, more knowledge is needed about the meaning of their living conditions, and how significant they are to the girls’ health. In an earlier study, Larsson, Johansson Sundler and Ekebergh (2012) found adolescent girls’ health to be a complex phenomenon interwoven into their everyday lives. Health and well-being were developed in meaningful situations in the adolescent girl's relationships to others as well as in their ability, which varied for each individual, to manage everyday life. The movements against being well were promoted in contexts that challenged adolescent girls to live and act out their lives, both physical and existential (Larsson et al., 2012). According to Ricœur (1992), humans live with a unique identity that develops in the community and in interactions with others. Human development, according to Mugerauer (2010), is affected partly by conditions present in biology and partly by the surrounding environment.

The importance of personal living conditions in the development of an adolescent girl's health and well-being seems to be an unexplored area. The aim of this study was to describe the meaning of living conditions in relation to adolescent girls’ health.

## Method

The study follows the principles of reflective lifeworld research (RLR), based on phenomenological and hermeneutic epistemology, as described by Dahlberg, Dahlberg, and Nyström (2008). The RLR approach builds on an interest in the lifeworld, which forms a foundation for understanding human experiences. This approach emphasizes openness to the phenomenon being studied. To be open means that the researcher's attention must be directed toward the studied phenomenon (Dahlberg et al., 2008). Empirical data consisting of 15 interviews collected by the first author (ML) in 2010 focusing on adolescent girls’ health were used.

### Description of participants and data

The participants were aged between 13 and 19; they all lived in western Sweden in areas ranging from mid-sized communities to rural. The girls’ family situations varied in that many had siblings, some lived with both parents, others with their mother, and some alternated between their mother and their father. Some of the girls had separated parents who had new partners. One girl lived alone. Half of the girls went to public school, 7th to 9th grade, and the other half went to secondary school, enrolled in either a theoretical or vocational educational program. One girl had previously lived in other countries in and out of Europe, and another girl had a parent with a different cultural background. The native language of all the girls was Swedish.

The participants decided on a date and place for the interviews, and all interviews were conducted in private rooms. Qualitative interviews (Kvale, Brinkmann, & Torhell, 2009) were conducted to enable personal disclosure focusing on adolescent girls’ lived experiences of health in everyday life. The interviews started with the question: “Can you tell me about your everyday life?” This initiated a dialog and enabled researcher and participant to become acquainted, where the participants were encouraged to narrate, with examples, of their experiences of health in everyday life. With the aim of being open, questions were formulated in the interviews depending on the situation. Follow-up questions were used to initiate reflections on the participants’ health (Dahlberg et al., 2008), for example, How do you mean? How did you feel then? The interviews, which were recorded and transcribed verbatim, lasted between 30 and 80 min, averaging 50 min.

### Analysis

The analysis was guided by the principles for reflective lifeworld research (Dahlberg et al., 2008). A hermeneutic interpretive analysis was performed to describe the meaning of living conditions in relation to adolescent girls’ health.

First, all data were read with a focus on the meaning of personal living conditions for health experiences. Meaning units were sought and compared to identify similarities and differences. As the analysis proceeded, certain living conditions and their meanings were gathered in patterns. Patterns descriptive for the girls were then structured into themes. The themes were put together in four narratives, the writing which was guided by the method for narrative configuration described by Polkinghorne (1995). This means that events, happenings, and actions are put together and integrated into a temporally structured whole.

Second, the analysis became more interpretative. In this phase, the analysis focused on how the girls experienced their living conditions and how these experiences influenced the girls’ health in everyday life. Reasonable interpretations that could clarify the girls’ health and living conditions were searched for and formulated in tentative existential interpretations. Ödman (2007) proposed that an existential interpretation is directed toward meaning, suggesting how humans understand their situations in the world. Tentative existential interpretations and their validity were evaluated in accordance with Ödman's (2007, p. 120) criteria. First, the source of a valid interpretation should be actual pieces of data. If the interpretation leaves a considerable amount of data unexplained, then the interpretation is viewed as weak. Second, no other interpretations could be found that more implicitly explain the same data, and third, there must be no contradictions in the data supporting an interpretation that is considered valid.

Finally, when the tentative existential interpretations were deemed valid, the analysis proceeded. The existential interpretations were compared and examined to interpret a pattern in the meanings. To deepen the understanding of living conditions in relation to adolescent girls’ health, existential interpretations were developed based on the lifeworld dimensions described by Merleau-Ponty (2002) and van Manen (1997). This resulted in a comprehensive understanding of all data of general importance for the phenomenon, which can be seen as an interpreted whole. To uphold an open attitude, the researchers discussed the findings from the interviews during the analysis, and circled between the parts and the whole of the text when discussing the tentative interpretations. Through the analysis, the researchers strived to question their pre-understanding and compared the interpretations of the interview contents, so that the researchers’ pre-understanding did not control what appeared in the data (Gadamer, 2004).

### Ethical considerations

Written informed consent was obtained from all participants and from the guardians of the participants who were under 15 years, in accordance with Swedish law. To protect the anonymity of the participants in the findings, narratives present a summary of the data instead of quotations. The narratives have fictitious names for the girls. All of the narratives consist of meaning from more than one participant. The study was conducted in accordance with the Helsinki Declaration and was approved by the ethics committee in Gothenburg, Sweden (dnr: 744-09).

## Result

The result consists of four narratives with their existential interpretations illustrating different ways of approaching living conditions and their meaning for health and well-being. The narratives are: *Approaching everyday life in a balanced way—feeling harmonious, approaching everyday life with ambiguity—feeling confused, approaching everyday life as an intellectual project—striving for control, and approaching everyday life as a struggle—feeling forlorn*. Subsequently, the comprehensive understanding follows.

### Approaching everyday life in a balanced way—feeling harmonious

Mia lives with her parents and siblings. She describes her family as caring and being there for her; she feels that her mother listens to her, and that she can talk with her parents about anything. Among her friends, she has a well-known core group, and she continuously builds new relationships with others who become her friends, both in and out of school. She enjoys playing sports that challenge her. When in a team, she feels togetherness when contributing and playing together with others. School is a natural part of her everyday life where Mia wants to learn new things. Sometimes she finds the time spent in school to be boring, depending on whether she experiences the school as too tough, even if demanding schoolwork also motivates her learning. When something worries her or makes her downhearted, she handles her feelings through conversations with others or by reflecting on the things that have happened. Mia is often happy and enjoys living her life, without feeling it to be bothersome or strenuous.

#### Existential interpretation

Mia's living conditions are characterized by balance and harmony. She seems to have stability in the relationships with her family and her close friends, as well as a sense of self-confidence. Community with others and being in meaningful contexts can be understood as crucial for the balance in her everyday life. The balance and confidence seem to give Mia a safe and secure background for her growth and future development.

### Approaching everyday life with ambiguity—feeling confused

Sue is in the middle of her teenage years. Her parents are divorced and with her siblings, she lives one week with her mother and every other week with her father. In her early teenage years, Sue was shy and lonely, without friends. A friend she found with similar experiences helped Sue change, and now she is curious about others. She has friends she cares about. Sue enjoys being in school and in teams. Sue describes herself as moody: one moment she is happy, and in the next she is bad-tempered or sad. She has difficulties handling her mood changes, and she is afraid that they affect her friendships. She often becomes irritated with her mother or siblings where these relationships are often conflict-ridden, which she demonstrates through hot-tempered language. She shares a hobby with her father and they do it together. She enjoys being with her friends, but also needs to be by herself. When on her own, she relaxes through listening to music, reading books, or watching TV.

#### Existential interpretation

The personal circumstances of Sue's everyday life seem to be confusing and frustrating for her. Even if her life appears balanced, Sue may experience ambiguity between the person she wants to be and not wanting to be a victim of her mood swings. She seems to want to take responsibility for herself and her life, even if she sometimes does not know how to go about it. She can be understood as uncertain concerning choices and how to handle everyday life. The ambiguous circumstances in life seem to take a lot of energy for Sue, and it becomes demanding for her to manage her life while she wants to take care of relationships that are meaningful and important to her.

### Approaching everyday life as an intellectual project—striving for control

Lena is in the middle of her teenage years and lives with her parents and siblings. Lena has high demands and expectations of herself. She feels that her parents, teachers, and others of the same age at school also have high expectations of her. She is ambitious, and when she succeeds, she feels she has support from those around her; in contrast, when she performs poorly, she misses this support. She makes detailed plans to reach her targets, especially in school; she is very focused and plans her time in detail. Lena likes discussions, and she wants to have knowledge and find arguments. Few of her classmates or friends have high ambitions like her, and sometimes she even gets angry with them for interfering or disturbing her educational pace. Even if she wants to have friends to share her interests with she finds it difficult and she rarely takes time out to simply to have fun. Lena often thinks that she disturbs her friends and that she is wasting their time. She has experiences from moving frequently in her childhood and memories of old friends who have forgotten about her, as she has gradually been forgetting them with the passage of time.

#### Existential interpretation

Lena seems to view her life as an intellectual project. She analyses and makes detailed plans for her life but seems to avoid personal disclosure and feelings. Her experiences from leaving close friends behind can be understood as the basis of her emotional wounds. Lena seems to strive for control of herself and her feelings. By distancing herself from feelings, both her own and others, she seems to live her life as a contradictory life project. Her relationships with others appear confusing, as she seems to avoid emotional contact while also seeking it. While living her life as an intellectual project, she seems to be betraying herself. She misses togetherness with others, and appears to replace it with something else.

### Approaching everyday life as a struggle—feeling forlorn

Sara is a young adolescent girl living with her mother and her mother's new partner, seeing her father sporadically. Earlier Sara experienced her mother being there for her whereas Sara now often feels that her mother acts unsympathetically and in a controlling fashion, with an attitude of “knowing best”. Their relationship is conflict-ridden. Sara has been let down several times by others she trusted and is cautious in relationships. Sara has a tough attitude and has been in conflict with some of her classmates, who avoid her so she is excluded from their community. The time she spends in school, which she does not like, is problematic. She has few friends, finds the subjects hard and has failed some of them, and thinks the teachers and the lessons are boring. Sara sometimes visits the school nurse or the welfare officer to have somebody to talk to about her situation, somebody with whom she dares to share her experiences and gain support. Sara sometimes spends her free time with some friends at the youth club, but mostly spends time with her horse, which she loves to take care of.

#### Existential interpretation

Sara's living conditions appear to make her feel forlorn. She seems desolate, missing community and relationship with others, especially her mother. Her everyday life can be understood as a struggle. Her weak self-confidence and her failures in relationships seem to make her fight for herself and for her existence. Her life has come to be about protecting and defending herself. Her tough attitude creates an image of being fearless while on the contrary, she seems to be vulnerable and exposed in her loneliness.

## Comprehensive understanding

The existential interpretations in the four narratives show how different aspects of living conditions seem important for adolescent girls’ health and well-being. To further deepen our understanding of the meaning of living conditions in relation to adolescent girls’ health, lifeworld dimensions, as developed by Merleau-Ponty (2002) and van Manen (1997), have been used. These four dimensions are: lived body, lived space, lived time, and lived relations. These dimensions are fundamental for how humans experience the world.^1^ By using them, complex living conditions have been explored, and the meaning of different parts clarified. With the help of the lifeworld dimensions, puzzle pieces are created that enclose different living conditions. The shape and size of the puzzle pieces seem to vary, and depending on how they are put together, different patterns emerge. Every adolescent girl's experiences of her health can be understood in light of the pattern she carries. Her health needs to be understood against the background of her experiences of her living conditions, in which the lived body, time, space, and relations seem to be important parts.

The dimensions can be understood as interwoven in experiences, but in the analysis, the dimensions have been distinguished for the nuances of the experiences to be elucidated.

### Lived body

The adolescent girl seems to try and challenge her body by acting and taking place in space, to explore who she is. Depending on what she encounters, she creates a picture of herself and how she presents herself. van Manen (1997, p. 103) said: “In our bodily presence we both reveal something about ourselves and we always conceal something at the same time.” Living conditions that are supportive and permissive seem to support development and well-being. The lived body feels. Todres, Galvin, and Dahlberg (2007, p. 57) describe it as the “bodily self ‘melts’ with love or tense up with fear.” Emotional expressions are versatile and simultaneously both controllable and uncontrollable, which seems to complicate being in a shared world with others. Adolescent girls seem to search through the lived body, in various ways, to participate in meaningful contexts.

### Lived space

The environments in which the girls move between seem to be valued based on the environments’ relevance to everyday life. Furthermore, the world around the girls seems not to have one unequivocal meaning but several possible meanings that vary depending on how they fit into the current situation of the girl's life. van Manen says “the space in which we find ourselves affects the way we feel” (1997, p. 102). For example, school influences adolescent girls’ health in ways which depend on the experiences the girls have in those lived rooms and how those experiences can be handled. van Manen describes that “lived space is the existential theme that refers us to the world or landscape in which human beings move and find themselves at home” (1997, p. 102). A girl's movement between environments seems to depend on how she experiences relationships with things and others in the environment, irrespective of the distance.

### Lived time

The girls’ previous experience and the context in which they live seems to shape their present lives. Merleau-Ponty (2002, p. 478) says that “what is past or future for me is present in the world.” The girls live their everyday lives within the background of experience; their previous experience is simultaneously altered by the impact of the present. A narrative can be created when a lived time provides a setting for experiences. In the narrations, the girls’ beliefs about the future are intertwined with their present and their past experiences, whether positive or negative. van Manen (1997, p. 104) proposes that “through hopes and expectations we have a perspective on life to come, or we may have lost such perspective.” The girls seem to struggle to find parts in their everyday lives that they experience as meaningful, such that they can be understood to be near the breaking point of what they can cope with.

### Lived relation

Being part of the family appears to be important for girls, which includes their close friends. In relationships with others, the girls seem to be aware of themselves and how they contribute to, and are influenced by, the context. van Manen (1997, p. 104) proposes that “as we meet the other, we approach the other in a corporeal way: though a handshake or by gaining an impression of the other in the way he or she is physically present to us.” The girls are influenced by what they encounter in many ways, including through body language, gestures, vocabulary, and tone of voice. How the girls are met and what they communicate in encounters with others seems to affect the possibility of being in a community as the person they are. Relationships with others instill feelings of confidence that strengthen the girls so their existence stabilizes, and thus is perceived as safe. When relationships with others do not instill confidence, the girls look for other ways to be safe such as for a community that gives meaning to life.

## Discussion

The results show that an adolescent girl's living conditions are meaningful aspects that influence her health and everyday life. These parts can also make the girls feeling: harmonious, in control of, confused or forlorn. The girls’ thoughts and feelings are often ambiguous and sometimes contradictory. In this study, all the participants were similar superficially in that they lived within families and went to school, which can be perceived as their everyday life. Nevertheless, their living conditions had different meanings and affected their health in varies ways.

The results show that for adolescent girls, relationship with others can be understood as both a possibility for them to feel well, as well as a basis for feelings of loneliness. It seems to depend on which meaning it had for the girl in her current life situation. In adolescence when a girl strives to be independent and free herself from their parents (Allen & Miga, 2010; Hwang & Nilsson, 2011), the relationship she has with her parents and friends seems to be important for her health. Relationships in which girls can be themselves, instill feelings of confidence and stabilizes their existence. Our results is in accord with what Landstedt (2010) means are important for adolescents girls mental health and what Kostenius and Öhrling (2008) found mutual relationships to be for children's well-being. Our results show that not all adolescent girls have this kind of relationship with their parents and friends, which is in agreement with other research (Currie et al., 2008; Statens Folkhälsoinstitut, 2011). In our study, the girls seemed to need somewhere to talk with somebody about everyday life as a way to enable well-being. The places need to have a low threshold for talking about health, as for example with the school nurse (Golsäter, Sidenvall, Lingfors, & Enskär, 2011; Langaard & Toverud, 2009). It seems important to have places where they can discuss health issues in a respectful and open atmosphere (Golsäter, Sidenvall, Lingfors, & Enskär, 2010), and where they can talk about their everyday life and what it means for them at the moment. These are places where the caregiver needs to be available and have time for individual meetings (Morberg, Dellve, Karlsson, & Lagerström, 2006) and make the girls feel that they are participating (Lindqvist, Kostenius, & Gard, 2012).

In Sweden, school nurses invite pupils at 10, 13 and 16-years of age to an individual health dialogue. The school nurses work with a health and lifestyle questionnaire which is the basis for an individual health profile used as a tool in the health dialogues with the pupils (Golsäter et al., 2011; Socialstyrelsen, 2004). The health and lifestyle questionnaire focuses on issues such as school, family, sleep, alcohol as factors for a healthy or unhealthy life. The school nurses use the questionnaire as a structure for the health dialogues and as a starting point, focusing on individual aspects (Golsäter et al., 2011). The health and lifestyle questionnaire is based on risk factors and on ways of decreasing the risk of cardiovascular disease and diabetes mellitus (Golsäter et al., 2011; Lingfors, Lindström, Persson, Bengtsson, & Lissner, 2003; Socialstyrelsen, 2004). It may be a risk that the questionnaire guides the health dialogues between the school nurses and the adolescent girls, such that no space is given to what the girls think and feel and that they are deprived of a place to talk about their situation in the way they wants to. The girls also have the youth center, but they are mostly associated with sexual health (Sollesnes, 2010). It seems therefore that adolescent girls have few forums to discuss health and to get help. The participants in our study seemed to have difficulties in understanding the meaning of their own living conditions in relation to their health experiences. This means that caregivers, for example school nurses and nurses at primary health care centers, need to have an attitude of wanting to help the girls sort out experiences in different situations. If caregivers have the lifeworld dimensions as a given structure and work with open-ended questions that support reflection, it may be that hidden complex patterns can be made visible and the parts made understandable, for both the girl and the caregiver. Through reflections, the girl can be helped to be aware of her thoughts and feelings, how she acts in different places and everyday situations and to clarify where she is in balance or not, which can be a way of supporting her well-being.

The study was conducted in accordance with reflective lifeworld research, and a hermeneutic analysis made it possible to provide suggestions on how the material meanings in the data could be explored and understood. Creating narratives made it possible for the analysis of the events and situations to be combined in new wholes, in which the unique and specific could be preserved. Polkinghorne (2007) argues that the value of narratives is their ability to clarify and give insight into the phenomena with rich detailed and revealing descriptions.

The interpretations should be understood as interpretations related to the data as their origin and should not be taken as claims to truth. Throughout the study, the researchers sought to maintain an open position. Dahlberg et al. (2008) argue that to be open and responsive to the phenomenon in focus is essential for the research to be valid. The researcher's pre-understanding was regularly discussed during the study. Furthermore, an expert in the field who was not involved in the research reviewed the interpretations and findings.

## Conclusions and clinical implications

The health of adolescent girls needs to be understood against the background of their experiences of living conditions, in which the lived body, lived time, lived space, and lived relations seemed to be important parts. If caregivers, such as school nurses, use a health and lifestyle questionnaire, it should be used as a basis for a discussion about all aspects of the girl's everyday life, rather than as a checklist. Health promotion work that focuses solely on risk factors may lead to missing other important aspects that influences the adolescent girls’ health. One way to support adolescent girls’ health and well-being seems to be to supply them with forums where they can talk about their living conditions.

## Footnotes

1 The lifeworld is the world that people take for granted, the spontaneous acts that appear to be meaningful. Lifeworld is personal; humans live through interactions with others and the surroundings simultaneously. The personal and social world with others is inseparable and can be seen as mutually dependent. Humans influence and are influenced by the world they live in (Merleau-Ponty, 2002).

## Bios

**Margaretha Larsson**, RN, MSc, is a PhD candidate in caring science at Linnæus University, Växjö, Sweden.

**Annelie Johansson Sundler**, PhD, RN, is a senior lecturer in nursing at University of Skövde, Sweden.

**Margaretha Ekebergh**, PhD, RN, is a professor in caring science at Linnæus University, Växjö, and at the University of Borås, Sweden.
